# GIPR rs1800437 polymorphism: prevalence and possible associations with metabolic syndrome-related diseases in a Brazilian urban population

**DOI:** 10.1186/1758-5996-7-S1-A200

**Published:** 2015-11-11

**Authors:** Alexandro Marcio da Silva Mattos, Natasha Guimarães Ludwig, Gustavo Kendy Camargo Koga, Sarah Conchon Costa, Marla Karine Amarante, Tânia Longo Mazzuco

**Affiliations:** 1 Universidade Estadual De Londrina – UEL, Londrina, Brazil

## Background

Glucose-dependent insulinotropic polypeptide receptor (GIPR) is mainly found in the pancreatic beta cells but has systemic distribution and function. Some genetic GIPR variants were recently associated with obesity, diabetes and insulin resistance. Few studies had studied the genetic epidemiology of the GIPR, mainly in Europe.

## Objective

To determine the prevalence of a specific GIPR single nucleotide polymorphism (SNP) in a Brazilian city population and its association with anthropometric, socio-demographic and clinical characteristics including metabolic syndrome-related diseases.

## Materials and methods

This was an observational, descriptive and cross-sectional study. A total of 222 subjects (129 women, 93 men) were recruited from the University Hospital located in a Brazilian metropolitan area (approximately 1.067,214 inhabitants). Random stratification was performed considering gender and geographic regions (downtown, north, south, east, west and other metropolitan areas). Data were collected by personal interview including anthropometric and socio-demographic data and diagnosis for diabetes, hypertension and obesity (personal/family history). Genomic DNA was isolated from peripheral blood and GIPR SNP genotyping (rs1800437) was performed by PCR-RFLP. Data were analyzed using chi-square and odds ratio (OR), with significance level set at 5%. Two-way ANOVA was used to analyze differences between genotypes distributions and geographic regions. Mann-Whitney test was used for nonparametric variables.

## Results

The analyzed population was in Hardy-Weinberg equilibrium and the commonest genotype GG was detected in 162 subjects. The C mutant allele was found in 27% of the population studied, with higher prevalence in men (p=0.006; OR=0.44), in caucasians (p=0.0001; OR=0.28) and in hypertensive subjects (p=0.004; OR=0.40). In the north region, low prevalence of the C allele was observed (p<0.05). No significant associations were found between the SNP and body mass index, obesity, diabetes and family history for metabolic syndrome-related diseases.

## Conclusion

This study points to a potential role for rs1800437 in hypertension. Associations with gender and ethnicity were also found in this Brazilian population. Taking into consideration the rarity of the CC genotype, further studies in larger sample sets will be necessary to confirm these results.

**Figure 1:**
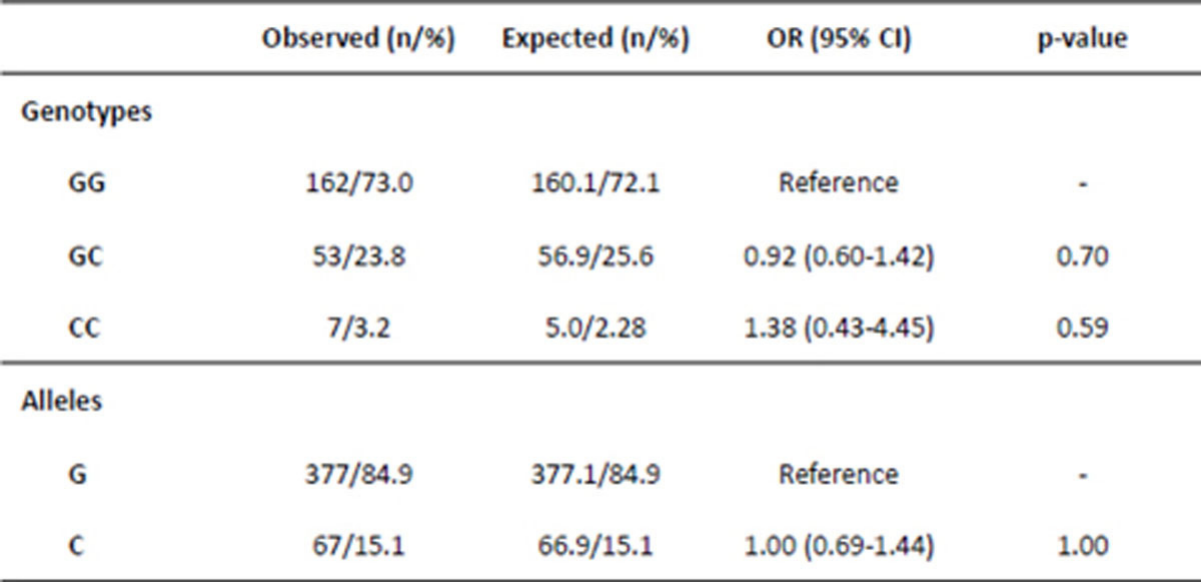
Hardy-Weinbeerg equilibrium for the GOPR SNP rs1800437 (Χ^2^-1.04, p=0.30)

